# Facts Relative to the Small Pox

**Published:** 1789

**Authors:** Thomas Davidson

**Affiliations:** Surgeon in Carriacou


II. *Facts relative to the Small Pox. Communicated in a Letter to Dr.* Simmons *by Mr.* Thomas Davidfon, *Surgeon in Carriacou.*

IN the month of January, 1786, upwards of fifteen hundred perfons, the greater number of whom were negroes, were inoculated for the fmall pox upon this ifland; and at that time a negro woman, then in the third or fourth month of her pregnancy, underwent the diſeaſe.

She was inoculated on the 11th of January, 1786, and was delivered of a girl about the ſame time of July following. This negro girl, when near three years of age, was inoculated in both arms on the 10th of May, 1789, with fluid matter taken immediately from a perſon under the diſeaſe. Suppuration of the arms took place as uſual, and about the ninth day the eruptive fever commenced, which, three days afterwards, was ſucceeded by a kind eruption of ſmall pox, to the number of forty or fifty puſtules.

From this caſe it will appear evident that the ſmall pox, ſeizing the mother while pregnant, will not always be communicated to the child *in utero*; although there have been the moſt undoubted proofs of this having ſometimes happened.

Here is one example in the earlier months of utero-geſtation.. *Quære*, Are infectious diſeaſes more liable to be communicated in the latter months?

Another caſe occurred here, which, as being ſomewhat ſingular, I ſhall beg leave to mention to you.

A boy,

A boy, about five years of age, having been inoculated with variolous matter upon a cotton thread, his arms ſuppurated at the uſual time, but no fever or eruption enſued. This induced the ſurgeon who attended him to apply ſome freſh fluid matter to the ſurface of the inciſions which had been formerly made in his arms, and which were then pretty large.— The application of freſh matter produced no other effect than another ſuppuration, from which freſh matter was furniſhed, and with it ſeveral others were inoculated, who all had the diſeaſe correſponding to the time when the operation was performed. Some weeks afterwards this boy was infected naturally, and had a vaſt number of ſmall pox.

Here the variolous matter being applied to an inflamed ſurface, produced matter *ſui generis* as uſual, but was not abſorbed, and therefore did not produce the diſeaſe.

If this was really the caſe, it confirms an idea, ſuggeſted by ſome modern anatomiſts, that an inflamed ſurface is a bad abſorbing ſurface.

In our general inoculation it was obſerved, that the ſtrong and athletic had moſt fever, and conſequently a greater number of puſtules than the weakly or delicate, who had very little fever, and few ſmall pox.

Perſons of all ages, from four weeks to ſixty years, were inoculated ; and ſome women who were as far advanced as the ſixth month in their pregnancy. Several women alſo were inoculated who had children at the breaſt ; and it was remarked that theſe children had more puſtules than their mothers.

*Carriacou,*

June 12, 1789.

